# Fundamental insights in early‐stage inclusion body formation

**DOI:** 10.1111/1751-7915.14117

**Published:** 2022-07-13

**Authors:** Julian Kopp, Benjamin Bayer, Christoph Slouka, Gerald Striedner, Mark Dürkop, Oliver Spadiut

**Affiliations:** ^1^ Research Division Integrated Bioprocess Development Institute of Chemical, Environmental and Bioscience Vienna Austria; ^2^ Department of Biotechnology University of Natural Resources and Life Sciences Vienna Austria; ^3^ Novasign GmbH Vienna Austria

## Abstract

Early‐stage inclusion body formation is still mysterious. Literature is ambiguous about the existence of rod‐shaped protein aggregates, a potential sponge‐like inclusion body scaffold as well as the number of inclusion bodies per *Escherichia coli* cell. In this study, we verified the existence of rod‐shaped inclusion bodies, confirmed their porous morphology, the presence of multiple protein aggregates per cell and modelled inclusion body formation as function of the number of generations.

## Graphical abstract

Literature is ambiguous about the existence of rod‐shaped protein aggregates, a potential sponge‐like inclusion body scaffold as well as the number of inclusion bodies per *E. coli* cell. In this study, we verified the existence of rod‐shaped inclusion bodies, confirmed their porous morphology, the presence of multiple protein aggregates per cell and modeled inclusion body formation as function of the number of generations.

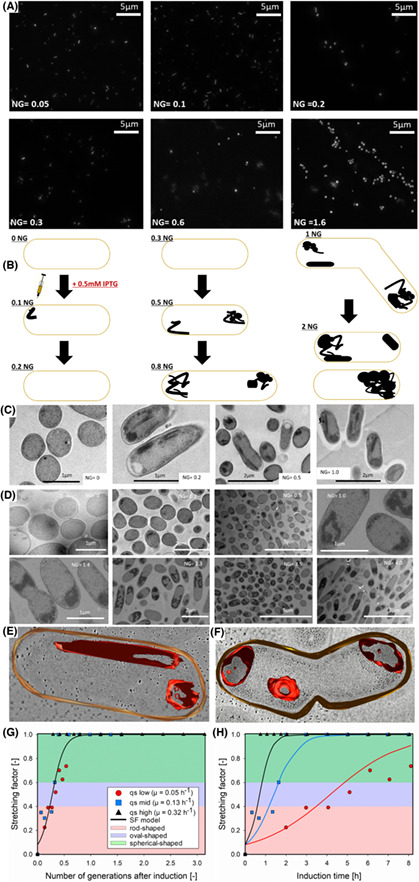

## INTRODUCTION

*Escherichia coli* is frequently used for the production of non‐glycosylated proteins due to its well‐characterized genome, cheap cultivation and fast growth (Rosano et al., 2019). However, high transcription rates of the target protein often lead to the formation of intracellular protein aggregates, known as inclusion bodies (IBs) (Lee & Hsu, 2018). IB formation can be reduced by either lowering the cultivation temperature during induction or by fusing solubility tags to the protein of interest. Still, the paradigm of IBs shifted and IB processing is applied in pharmaceutical industry up to date (Costa et al., 2014; García‐Fruitós et al., 2012; Rinas et al., 2017). The natural phenomenon of IB formation in *E. coli* has been known for decades (Fahnert et al., 2004; Rosano et al., 2019). In 2008, Lindner et al. established a model based on fluorescence microscopy data, describing the intracellular localization of native cell aggregates (Lindner et al., 2008). However, fundamental aspects of recombinant IB formation and IB characteristics remain either unknown or ambiguously discussed in literature due to the complexity of heterologous protein production and the mechanisms involved (Baneyx, 1999; Carrió & Villaverde, 2003, 2005). Moreover, the type of expression system as well as the age of the culture have major influences on protein aggregate formation (Baneyx & Mujacic, 2004). Combining these factors with a decreasing production rate for ageing cells may lead to various different IB agglomerates (Baneyx & Mujacic, 2004; Lindner et al., 2008). Lee and Hsu described IB formation as a three step‐reaction: partially misfolded peptide chains fuse to so‐called proto‐aggregates, which then merge to spherical IBs (Lee & Hsu, 2018). The existence of rod‐ and spherical‐shaped protein aggregates has been postulated, but an explanation of distinct IB morphologies is still missing in literature (Bowden et al., 1991; Carrió et al., 2000; Humer & Spadiut, 2018). We hypothesize the lack of proper analytical techniques to be the reason therefore. Pre‐IBs are presumed of a native‐like secondary structure representing germs for later IB formation (Oberg et al., 1994). Scanning electron microscopy (SEM) is frequently used to monitor IBs; however, IB samples are commonly prepared on a gold‐sputtered polycarbonate membrane (Carrió et al., 2000; Slouka et al., 2018; Wurm et al., 2018). Hence, SEM is not capable of monitoring early‐stage IB formation as (i) either the filter pore diameter exceeds the size of IB proto‐aggregates (Kopp et al., 2018; Slouka et al., 2018), or (ii) IB attachment onto the gold membrane could potentially trigger agglomeration between sample particles (Carrió et al., 2000; Kopp et al., 2018; Peternel et al., 2008), altering IB morphology, whereas atomic force microscopy showed a dominant fraction of spherical IBs (Sanagavarapu et al., 2019), confocal microscopy indicated spherical and cylindrical shaped IB‐structures inside *E. coli* cells (Gil‐Garcia et al., 2020). Historically, IBs are described as dense protein aggregates; however, recent cryo‐transmission electron microscopy (TEM) analyses indicated a sponge‐like scaffold (Cano‐Garrido et al., 2013; Ramón et al., 2014). Even though FTIR (=Fourier‐transform infrared spectroscopy) is a powerful tool to monitor protein folding (Miller et al., 2013), the concentration of initial IBs might be too small to monitor folding effects at this stage. Furthermore, current findings about the presence of multiple protein aggregates per *E. coli* cell are ambiguous (Peternel & Komel, 2011). Finally, only scarce modelling approaches on IB kinetics can be found in literature, even though bioprocess modelling has been proven to enhance the understanding and description of biological phenomena.

## EXPERIMENTAL PROCEDURES

### GFP ^+^ expression

GFP^+^ belongs to the group of auto‐fluorescent proteins and has 100% match with (Scholz et al., 2000). The gene of GFP^+^ was inserted into a pET21a^+^ vector using *Nde*I and *Xho*I restriction sites. The plasmid was transformed into competent *E. coli* BL21 (DE3) cells (Life Technologies, Carlsbad, CA, USA). GFP^+^ was reported at a protein size of 26.9 kDa and an isoelectric point of 5.9 (De et al., 2009). Estimated GRAVY index for hydrophobicity resulted in −0.467 and the aliphatic index was at 79.04.

### N‐pro protein expression

N‐pro protein has been reported as sufficient N‐terminal‐tag to increase protein hydrophobicity facilitating IB processing. The gene of the N‐pro protein was inserted into a pET30a^+^ vector. The plasmid was transformed into competent *E. coli* BL21 (DE3) cells (Life Technologies, Carlsbad, CA, USA). N‐pro protein has a protein size of 28.76 kDa and an isoelectric point of 8.78. Calculated GRAVY index for hydrophobicity resulted in −0.684.

### Bioreactor cultivations

All bioreactor and preculture cultivations were carried out using a defined minimal medium according to (DeLisa et al., 1999), with media details stated in Table S1. Batch and preculture media were supplied with glucose: 8.8 g/L for preculture and 22 g/L for batch media. Feeds were prepared with a concentration of 400 g/L glucose (DeLisa et al., 1999). Ampicillin was added to preculture and batch media in a final concentration of 0.02 g/L. Precultures were done in 500 ml high yield flasks. They were inoculated with 1.5 ml of bacteria cryo stocks and subsequently cultivated for 16 h at 230 rpm in an Infors HR Multitron shaker (Infors, Bottmingen Switzerland) at 37°C. All cultivations were either performed in a Sartorius Biostat Cplus bioreactor (Sartorius, Göttingen, Germany) with 10 L working volume (wV). Batch phase was conducted for 9 h, until a biomass of 10 dry cell weight (=DCW) g/L was received. From a non‐induced fed‐batch phase, we obtained 25 g/L DCW. Process parameters then were adjusted to targeted conditions. Induction was performed via a one‐point IPTG pulse, resulting in an IPTG concentration of 0.5 mM. To compare cultivations, the number of generations after induction of recombinant protein synthesis (= NG) was used. Pre‐results (Wurm et al., 2016, 2018) indicated that the ratio of IB to soluble protein formation is highly dependent on the NG and the cultivation temperature, favouring higher NGs and higher temperatures. Hence, we performed all cultivations at 35°C and investigated the pathway of IB formation at different NGs. Detailed cultivation information is given here (Kopp et al., 2018).

### IB preparation for microscopy imaging

Scanning electron microscopy and HRFM images were taken using washed IBs after cell disruption. Thereby, 5‐ml fermentation broth samples were centrifuged at 3100 *g* at 4°C. The supernatant was discarded, and the pellet was resuspended to a DCW of about 4 g/L in lysis buffer (100 mM Tris, 10 mM EDTA at pH = 7.4). Afterwards, the sample was homogenized using a high‐pressure homogenizer at 1500 bar for 3 passages (EmulsiflexC3; Avestin, Ottawa, Canada). After centrifugation at 6400 *g* and 4°C, the supernatant was discarded and the resulting IB pellet was washed twice with ultrapure water and aliquoted into pellets à 2‐ml broth, centrifuged (9000 *g* 10 min 4°C) and stored at −20°C. Detailed information on sample preparation can be found here (Slouka et al., 2018).

Transmission electron microscopy images were captured from intact cells, using fresh cultivation broth, thereby samples were taken according to the desired NG and prepared and frozen immediately for subsequent transmission electron microscopy.

### Scanning electron microscopy

Scanning electron microscopy was performed using a QUANTA FEI SEM (Thermo Fisher, Waltham, MA, US) with a secondary electron detector. The acceleration voltage of the electron beam was set between 3 and 5 kV. To determine the diameter of the IBs, 50 IBs on SEM pictures were measured using the ImageJ plugin Fiji (Laboratory for Optical and Computational Instrumentation [LOCI], University of Wisconsin‐Madison, US). Sample preparation required fixing an IB suspension in ultrapure water on a gold‐sputtered polycarbonate filter, with details given here (Slouka et al., 2018).

### High‐resolution fluorescence microscopy (HRFM)

Structured illumination super‐resolution microscopy was performed on a DeltaVision OMX V4 imaging system (GE Healthcare). 488, 561 nm lasers were used to excite the samples. Three angles and five phases were acquired for each surface. The raw images were reconstructed with the OTF (optical transfer function) of the same emission wavelength. IB pellets were dissolved in ultrapure water, placed on a slide and quantified directly in suspension.

### Transmission electron microscopy

#### Cryo‐preparation of *E. coli*

A pellet resulting from centrifuged fresh *E. coli* cells was mixed 1:1 with 20% BSA in a 0.9% NaCl buffer solution and transferred into the 100 μm cavity of a 3 mm aluminium specimen carrier. This carrier was sandwiched with a flat 3‐mm aluminium carrier and immediately high‐pressure frozen in a HPF Compact 01 (Engineering Office M. Wohlwend GmbH, Sennwald, Switzerland). The frozen samples were subsequently transferred into a Leica EM AFS‐2 freeze substitution unit (Leica Microsystems, Vienna, Austria). Over a period of 4 days, samples were substituted in a medium of acetone containing 2% Osmium tetroxide. (Freeze substitution was performed according to the following protocol: 30–40 h at −90°C, warm up at a rate of 2°C per hour to −54°C, 8 h at −54°C, warm up at a rate of 3°C per hour to −24°C, and 2 h at 0°C warm up at a rate of 2°C per hour to 0°C). At 0°C, samples were taken out and washed three times in anhydrous acetone (on ice) and infiltrated with Agar 100 Epoxy resin (Agar Scientific, Essex, UK), in a graded series of acetone and resin over a period of 3 days. Polymerization took place at 60°C. Ultra‐thin sections with a nominal thickness of 70 nm were cut using a Leica UCT ultramicrotome (Leica Microsystems, Vienna, Austria) and picked up on 100 mesh Cu/Pd grids previously coated with a supporting film of formvar. The sections were post‐stained with 2% aqueous uranyl acetate and Reynold's lead citrate. Examination regions on the sections were selected at random, examined with a FEI Morgagni 268D (FEI, Eindhoven, The Netherlands) operated at 80 kV. Digital images were acquired using an 11 megapixel Morada CCD camera (Olympus‐SIS, Muenster, Germany).

#### Electron tomography

For room temperature, electron tomography sections of a nominal thickness of 200 nm were made on a Leica UCT ultramicrotome (Leica Microsystems, Vienna, Austria). The sections were collected on a 50 mesh Cu/Pd grid (Gilder Grids, Lincolnshire, UK), previously coated with a supporting film of formvar, post‐stained with 2% aqueous uranyl acetate followed by Reynold's lead citrate. For tomogram alignment, 10 nm gold (Aurion) beads were put onto both sides of the section by incubating the grid in a drop of concentrated gold solution for 3 min. Tilt series were acquired at a Tecnai G2 20 microscope (FEI, Eindhoven, The Netherlands) equipped with an Eagle 4 k HS CCD camera (FEI, Eindhoven, the Netherlands) and operated at 200 kV. Dual‐axis tilt series were collected with a tilting range from −60° to +60° each at 1° increment. For data acquisition, processing and modelling the IMOD software from the Boulder Laboratory for 3D Electron Microscopy of Cells, University of Colorado Boulder, was used.

**FIGURE 1:**
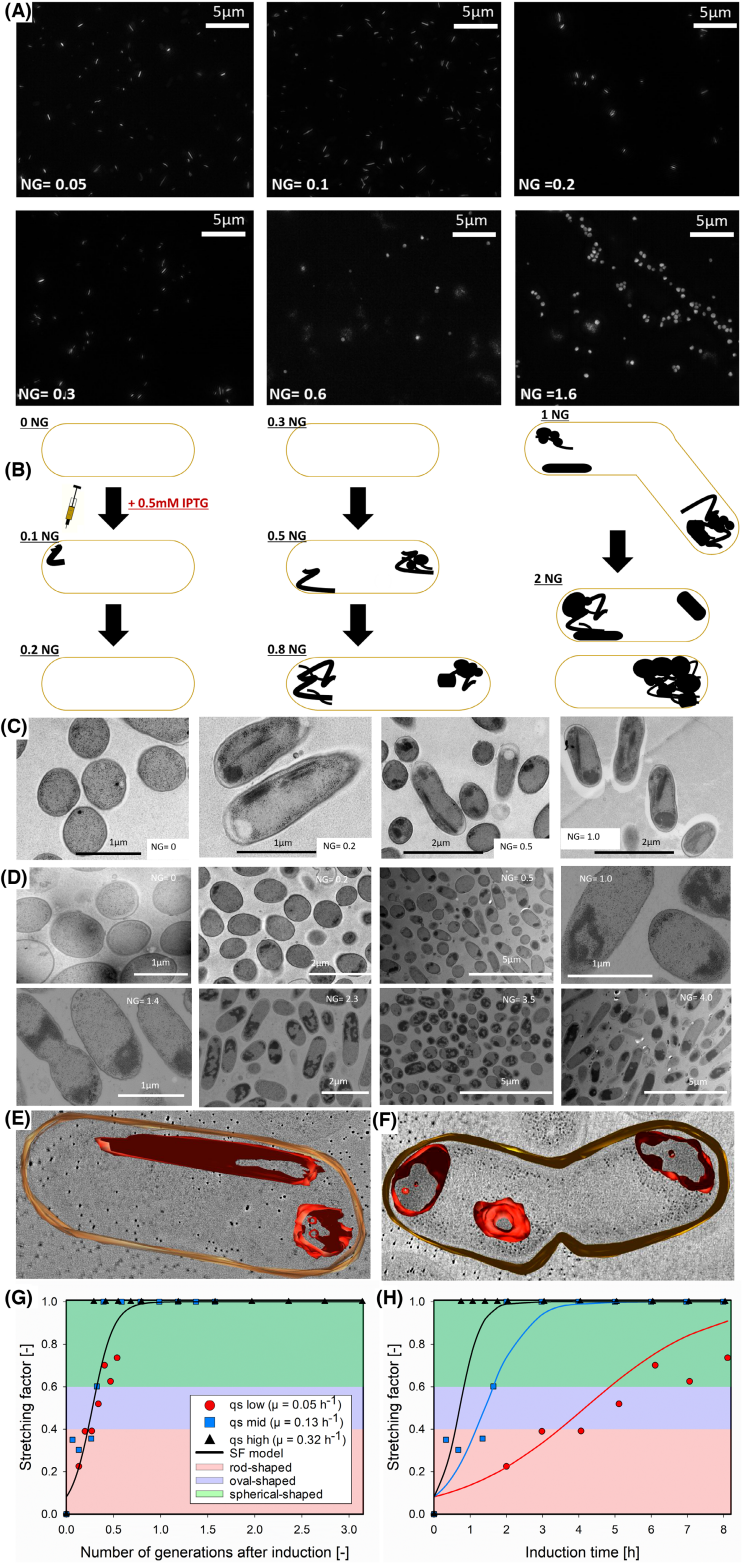
(A) High‐resolution fluorescence microscopy images of purified GFP IBs after cell disruption at different number of generations after induction of recombinant protein synthesis (= NG). Early‐stage rod‐shaped structures are present only until NG = 0.3, whereas at a later number of generations, only spherical IBs can be monitored; even though HRFM was able to measure only spherical IBs at a higher number of generations, we believe that rod‐shaped IBs are present at all stages of the process and that fluorescence of spherical IBs simply overlaps fluorescence of rod‐shaped IBs; (B) sketch of assumed IB formation: Rod‐shaped proto‐aggregates are formed after recombinant protein production was initiated. Rod‐shaped proto‐aggregates tend to agglomerate as a function of NG; (C) TEM images of the target protein GFP as function of the number of generations; in the reference sample prior to induction at NG = 0 no aggregates are formed, whereas recombinant protein is formed later on, rod‐shaped structures are formed present at all induction time points until NG = 1. Spherical IBs are formed at higher NGs potentially due to agglomeration of rod‐shaped proto‐aggregates; (D) TEM images of the target protein N‐pro protein as function of NG; rod‐shaped structures can be seen until NG = 4; after NG = 4, IBs of the N‐pro protein take up more than 50% of the cytosol and only 1 spherical IB can be detected; (E) electron tomography images of GFP IBs. (F) Electron tomography images of N‐pro IBs. (G) Process model to describe the change in the IB morphology as a function of the NG. The calculated SF values derived from the analytical measurements of the three GFP cultivations performed for different NGs after induction are shown as symbols, with q_s_ describing the specific substrate uptake rate applied in g/g/h. the alleged IB shape (rod: 0.01–0.20; oval: 0.21–0.50; spherical: 0.51–1.00), is indicated as coloured areas along with the SF model (solid line). H) Time‐resolved representation of the SF values from the analytical measurements and the model in GFP cultivations performed for different NGs. The comparative examination of the SF for the production phase highlighted the varying IB growth kinetics depending on the applied feeding rate.

Videos S1 and S2 result from cells prepared for TEM taking samples in 20 nm distances. By fusing all images of different microscopy heights, a densitometric model can be obtained showing the native structure of intracellular inclusion bodies. Videos S1 and S2 thus show the imaging at different heights, whereas Figure 1E,F shows the results of the densiometric model.

### Bioprocess modelling to characterize IB growth kinetics

The SF values (model output) were derived from the GFP cultivations (Figure 1A, Figures S3–S5) and defined as the ratio of the IB diameter and length as determined by HRFM (Figure S2), that is describing the IB shape from rod‐shaped (early‐stage) to spherical. To describe this *SF* as a function of the *NG* after induction (model input), a non‐linear least‐squares curve solution (logistic regression) was applied, and the used parameters were optimized to obtain the best fit (Equation 1).

(1) $$\mathrm{SF}=\frac{L}{1+e^{-k\left(\mathrm{NG}-\mathrm{NG}0\right)}}$$

where *L* represents the maximum value of the SF (1, spherical), *k* the slope (8.785), and *NG0* the NG value at which the curve reaches half of the maximum *L* value (0.2764). Complete process model development was performed with MATLAB (2020a, MathWorks, USA).

## RESULTS AND DISCUSSION

To shed more light on fundamental, early‐stage IB formation, we studied the well‐known reporter molecule green fluorescent protein (GFP) (García‐Fruitós et al., 2005) employing different microscopic techniques. GFP is known to form fluorescing (active) IBs (García‐Fruitós, 2010; Peternel et al., 2009), which can be monitored by high‐resolution fluorescence microscopy (HRFM). It was already shown that fluorescence microscopy is capable of monitoring a major increase in aggregate formation over the time of induction, using fluorescein bisarsenoxide labelled inclusion bodies (Ignatova & Gierasch, 2004). Even though the authors successfully applied fluorescent microscopy for these analyses, the IB morphology was not investigated within the scope of this study. To get more detailed information on the shape of IBs, we took HRFM images from pelleted IBs after cell disruption using an established washing and centrifugation protocol to separate aggregates from soluble fractions (Eggenreich et al., 2017; Palmer & Wingfield, 2012). Applying HRFM imaging techniques for purified IBs, we were able to prove the existence of rod‐shaped IB proto‐aggregates for the first time (Figure 1A). We compared HRFM data with SEM images of identical samples revealing the limitations of SEM (Figure S1), where only spherical IBs could be seen. Using timely resolved HRFM data of different cultivations performed at altered growth rates (Figure 1A, Figures S2–S5), we determined a correlation between the number of generations and the presence of distinct IB morphologies (Figure 1B). Growth rates for the conducted experiments were chosen based on the results of a pre‐study published in our group (Kopp et al., 2018). To compare cultivations as a function of cell division, the number of generations after induction of recombinant protein synthesis (= NG) was used. At low NGs, we observed only rod‐shaped IBs, whereas at higher NGs, only spherical IBs were seen. We thus hypothesize that nascent IBs are rod‐shaped and agglomerate to sphere‐like IBs as a consequence of cell division (Figure 1B). As literature describes IB size to increase with induction time (Kopp et al., 2018; Lindner et al., 2008), we assume that two IB‐related events happen during cell division: misfolded peptide chains either form de novo rod‐shaped proto‐aggregates or are deposited on existing rod‐shaped IBs forming spherical, porous IBs, commonly described as IB ‘growth’ (Kopp et al., 2018; Peternel et al., 2008). As GFP is forming active inclusion bodies, we assume that quantified early‐stage proto‐aggregates match the theory introduced by Peternel et al, stating the existence of soluble proto‐aggregates (Peternel & Komel, 2011). We believe that we could not detect rod‐shaped IBs using HRFM at NGs higher than 0.3 (Figure 1A), as the fluorescence gain of spherical IBs overlapped the fluorescence of rod‐shaped IBs. The ‘ghost‐like’ fluorescence at higher NGs of 0.6 and 1.6 correlates to the higher amount of IB present in the culture, thereby causing higher fluorescence by the laser excitation.

To verify whether rod‐shaped IBs were present at a NG higher than 0.3, we monitored intracellular IB formation as a function of NG using TEM (Figure 1C). Results proved that rod‐shaped structures were present until a NG of 1, whereas spherical IBs were only present from NG of 0.5 onwards (Figure 1C). Thus, TEM confirmed that rod‐shaped IBs agglomerate to spherical IBs, being in accordance with HRFM microscopy (Figure 1A).

We extended our study to the N‐pro protein (Achmüller et al., 2007), to prove the existence of rod‐shaped proto‐aggregates and test our hypotheses yet with another model protein. N‐pro formed bulkier protein rods (Figure 1D) compared with GFP (Figure 1C), which we attribute to the higher hydrophobicity of N‐pro (Achmüller et al., 2007). Still, we could successfully prove the existence of rod‐shaped proto‐aggregates also for N‐pro, which we monitored until a NG of 4 (Figure 1D). From there on, only spherical IBs could be detected. We hypothesize that a co‐existence of both IB morphologies is only possible if the spherical IB does not exceed a certain size. We attribute this phenomenon to a simple steric hindrance inside the cell and the deposition of misfolded peptide chains on the protruding spherical IB. In fact, at a NG, of 4 the spherical N‐pro IB covered more than 50% of the cytoplasmic space.

To strengthen our hypothesis that rod‐shaped proto‐aggregates agglomerate to porous spherical IBs, we investigated the IB scaffold in more detail. Govers et al. disassembled IBs of the yellow fluorescent protein using high hydrostatic treatment and observed a reassembly of these fragments to a porous IB (Govers et al., 2014). Cano‐Garrido et al. used cryo‐TEM to analyse IBs and described them as amyloid structures with a sponge‐like scaffold (Cano‐Garrido et al., 2013; Ramón et al., 2014). We extended TEM analysis to electron tomography analysis, allowing 3D imaging of IBs (Figure 1E,F). Videos S1 and S2 result from cryo‐preparation of *E. coli* (described in more detail in the Section 2) indicating that rod‐shaped aggregates (being observed as more rectangular aggregate‐like shape in Video S1) showed a more compact and dense structure compared with spherical IBs. By monitoring the intracellular IB composition in 20 nm distances, a densiometric model can be generated being shown in the supplementary videos, thus showing the native structure of intracellular inclusion bodies. We hypothesize that the higher porosity of spherical IBs occurs due to agglomeration of rod‐shaped proto‐aggregates collapsing upon cell division resulting in voids and pores in the resulting spherical IB (Figure 1E,F).

Existing IB models are based on IB data of fluorescent fusion proteins and only describe the cellular age as response of the fluorescence intensity, measured at cellular poles (Lindner et al., 2008). However, the morphological change of IBs is not yet mathematically described in literature, which is why we developed a process model to describe the maturing of IBs as a function of the NG (Figure 1B). We named this transition from rod‐shaped to spherical IB morphology the ‘stretching factor (SF)’. The established SF model is based on the HRFM GFP data (Figure 1A) to mechanistically describe the change in IB morphology. The derived model values for the SF match the calculated values utilizing the analytical measurements well (*R* ^2^ = 0.87). The model was fitted to describe data, that is the presence of rod‐shaped IBs at lower NGs and the subsequent formation of spherical IBs with increasing NGs (Figure 1G). By examining the derived analytical and model SF values over the entire induction time, the impact of the applied NGs on IB morphology throughout the different cultivations can be visualized (Figure 1H). However, the SF model was only established for GFP due to data availability at an early number of generations. To confirm its general applicability, further data of early IB formation need to be generated for different target proteins in future studies.

Apart from the established model, conducted TEM analyses did not only provide useful data on IB shape but also delivered information regarding the number of aggregates per *E. coli* cell. For the expression of many recombinant proteins, it is reported that one single spherical IB can be found per *E. coli* cell (Peternel & Komel, 2011), mainly located at a cell pole (Govers et al., 2014; Laloux & Jacobs‐Wagner, 2014). Confocal microscopy images showed that either large single IBs can be present in one cell or two smaller IBs per cell can be detected (Gil‐Garcia et al., 2020). Results in this study showed that multiple IBs can be present per *E. coli* cell (Figure 1C,D), being in accordance with high‐resolution TEM images presented here (Castellanos‐Mendoza et al., 2014). However, once IB size exceeds 50% of the cytoplasmic cellular volume, aggregates tend to fuse to one larger IB per cell (Figure 1D). We attribute this behaviour to hydrophobic interactions between aggregates, collapsing to larger IBs due to steric hindrance inside host cells.

Summarizing, this contribution proves the existence of rod‐shaped IB proto‐aggregates for the first time. Our findings indicate a steady formation of nascent rod‐shaped IBs during induction time, which agglomerate to spherical IBs with proceeding NG. Hence, we explain the reported porous scaffold of spherical IBs by agglomeration of rod‐shaped IB proto‐aggregates. This statement was further reinforced by a logistic bioprocess model, describing the formation of IBs as a function of the number of generations. A mathematical link between the NG of cells and distinct IB morphologies could be confirmed via the SF. Additionally, we could confirm the presence of multiple IBs per cell and thus fill fundamental knowledge gaps with respect to IB formation.

## AUTHOR CONTRIBUTIONS

JK and CS performed the cultivations and conducted data treatment. BB, GS and MD established the model and gave major scientific input. JK, CS and OS founded the idea of the study. JK, BB, GS, MD and OS wrote the manuscript.

## FUNDING INFORMATION

TU Wien Bibliothek; Österreichische Forschungsförderungsgesellschaft (Grant/Award Number: ‘874206’).

## CONFLICT OF INTEREST

The authors declare that they have no conflict of interest.

## Supporting information


Appendix S1

Video S1

Video S2

## Data Availability

The datasets generated and/or analysed during the current study are not publicly available as they were created throughout the course of a project with an industrial partner but are available from the corresponding author on reasonable request.
