# Supplementary material for: Fundamental insights in early‐stage inclusion body formation
Source: Microb Biotechnol. 2022 Jul 13;16(5):893–900. doi: 10.1111/1751-7915.14117 (PMC10128139; doi:10.1111/1751-7915.14117)
Supplement: Supplementary file 1 — Appendix S1 [file MBT2-16-893-s001.pdf]

Fundamental insights in early-stage inclusion body formation

Supplementary Material

J. Kopp[1,§], B. Bayer [2,3], C. Slouka[1], G. Striedner[2,3], M. Dürkop[2,3], O. Spadiut[1]

[1] Research Division Integrated Bioprocess Development, Institute of Chemical, Environmental and Bioscience Engineering

[2] Department of Biotechnology, University of Natural Resources and Life Sciences, Vienna, 1190 Austria

[3] Novasign GmbH, Vienna, 1190 Austria

§Correspondence and Requests for materials should be addressed to:

Julian Kopp, TU Wien, Institute of Chemical, Environmental and Bioscience Engineering, Research Division Integrated Bioprocess Development, Gumpendorfer Straße 1a, 1060 Vienna, Austria. Tel.: +43 1 58801 166485, Email: julian.kopp@tuwien.ac.at

Media composition

Supplementary Table 1: Composition of the defined medium which was used for all performed cultivations adapted from Delisa et al.

DeLisa Medium	final conc. [g/l]
Glycerol	22,00
KH_2PO_4	13,30
$(NH_4)_2HPO_4$	4,00
Citric acid	1,70

Trace-elements (prepared separately)	final conc. [g/l]
$MgSO_4$ * 7 H_2O	1,20
Fe(III) citrate	0,1000
EDTA	0,0084
$Zn(CH_3COO)_2$ * 2 H_2O	0,0130
$CoCl_2$ * 6 H_2O	0,0025
$MnCl_2$ * 4 H_2O	0,0150
$CuCl_2$ * 2 H_2O	0,0012
H_3BO_3	0,0030
Na_2MoO_4 * 2 H_2O	0,0025
Thiamine HCl	0,0045

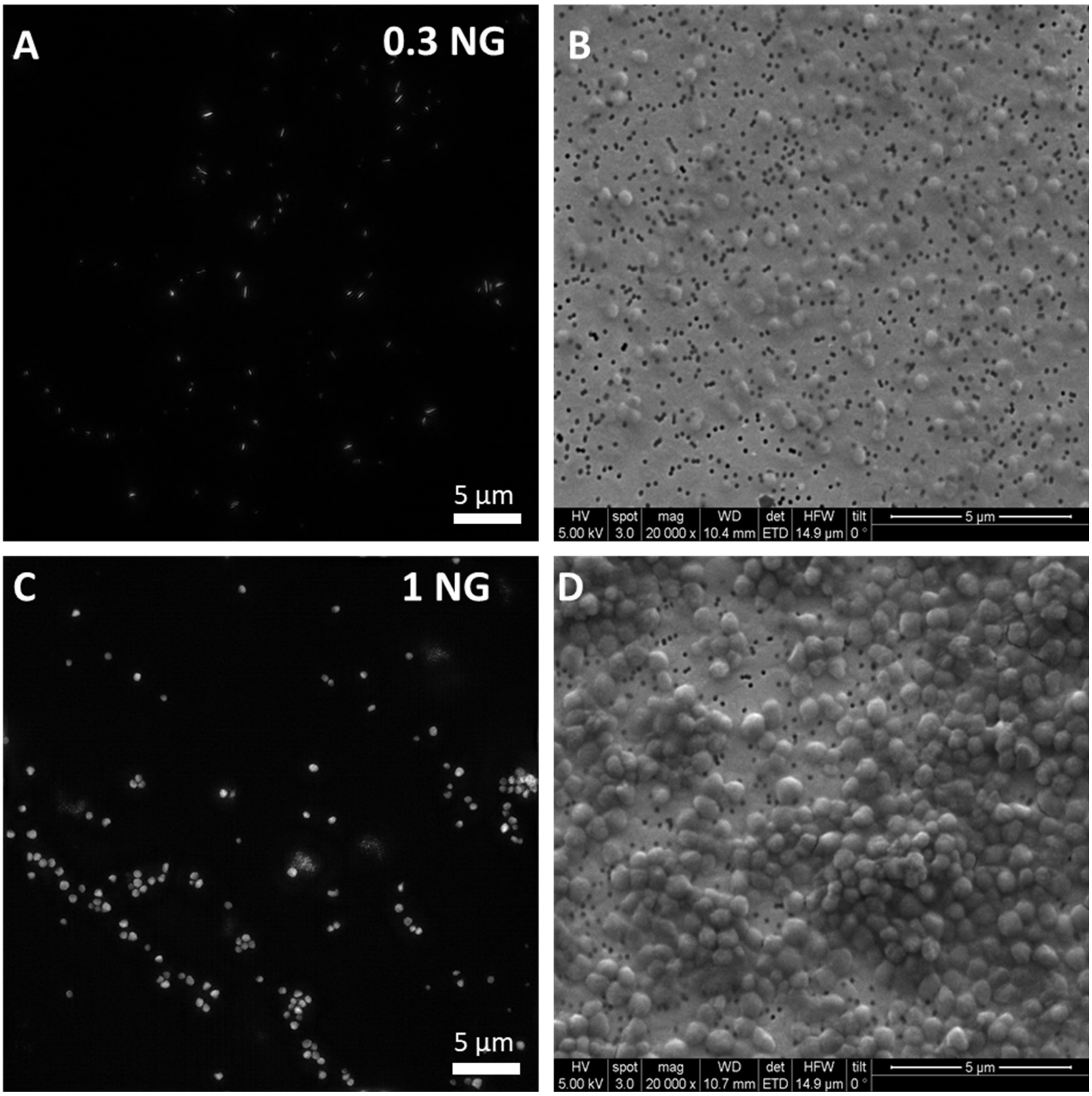

Supplementary Figure 1: Inclusion Body size analyzed with high resolution fluorescence microscopy (A & C) and with scanning electron microscopy (B & D). Samples in figure 1 A and B were taken after 0.3 number of generations after recombinant protein synthesis was induced (=NG) whereas Figure 1 C and D were samples at a NG= 1. Fluorescence microscopy is differentiating between sphere-like IBs (C) and rod-shaped IB structures (A). SEM pictures are limited to 2D structures, due to sample filtration onto polycarbonate filters, explaining the observed round structure. SEM samples after NG=0.3 (B) show a smaller IB diameter compared to samples taken at NG=1 (D)

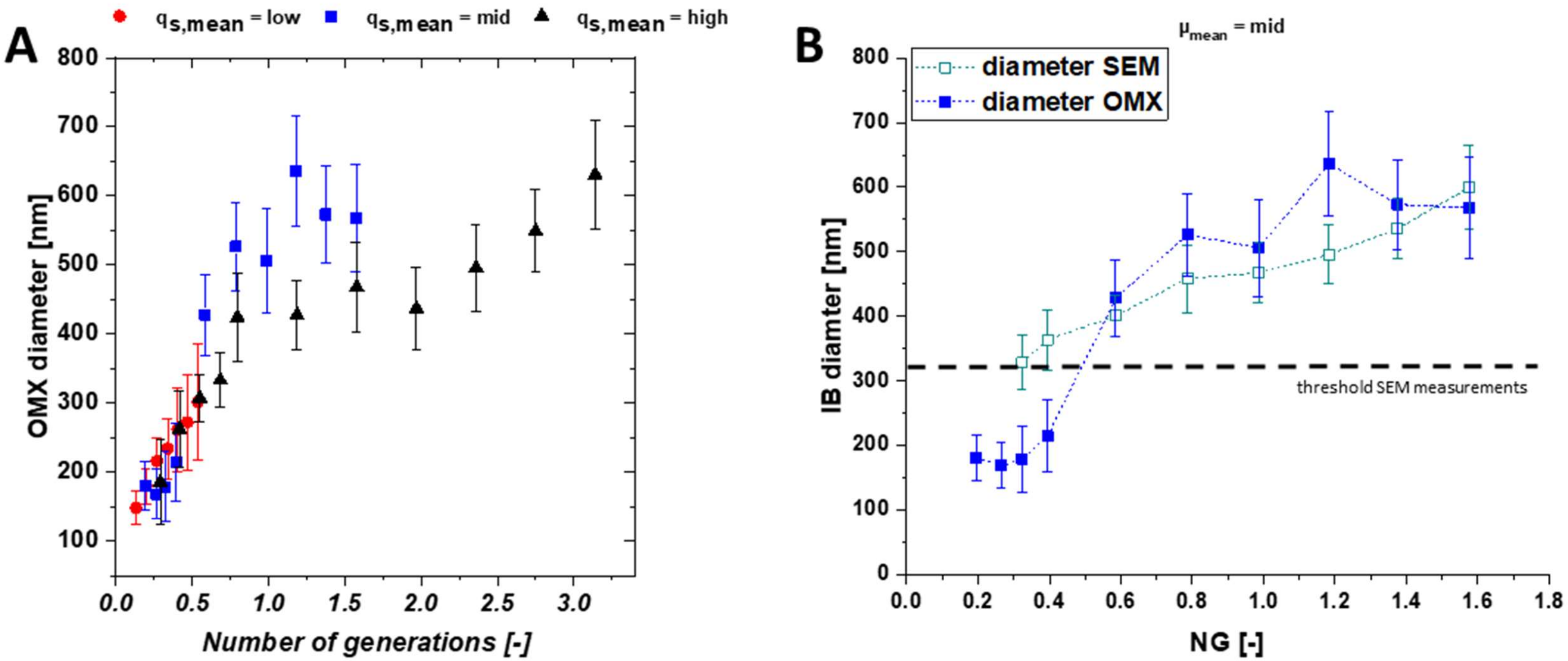

Supplementary Figure 2: A) Inclusion Body size analyzed with high resolution fluorescence microscopy and measured diameters received for three different cultivations. Thereby we can see a correlation of the doubling rate and the inclusion body size especially in early stages of IB formation (below 0.5 number of generations). Late stage inclusion body size might be more error prone as for OMX analysis strong fluorescent gains need to be applied to monitor the IBs. The emitted fluorescence around the IBs thus might influence the graphical evaluation of samples (i.e. contrasting of samples). B) Showing the differences between OMX and SEM measurements especially for measurements at low NGs, the OMX is more sensitive than SEM and also a major difference in size can be obtained comparing SEM and OMX at early stages

Supplementary Figures 3-5:

Supplementary figures 3-5 give an insight in morphology alteration of GFP-IBs expressed with different feeding rates. Results indicate that rod-shaped IB formation is visible up until NG ~0.5-0.7, with some spherical IBs present also. At a higher number of generations (NG >0.7) only sphere like IBs can be monitored via OMX. Results for high-fluorescence resolution microscopy images can be obtained for each individual cultivation within the next slides.

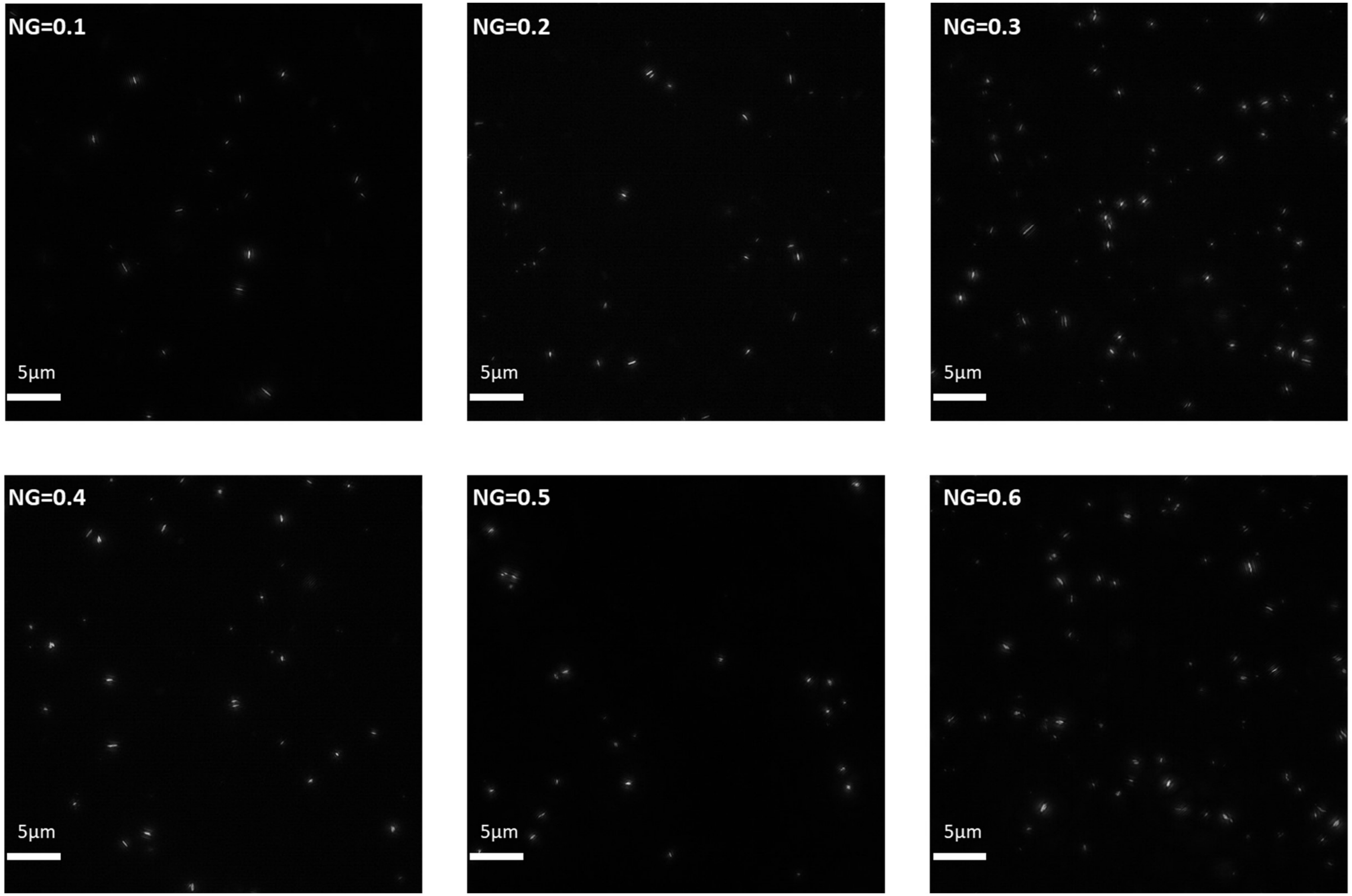

Supplementary Figure 3: Time-dependent OMX analysis of purified GFP IBs conducted for the cultivation operated at $\mu \sim 0.05\ h^{-1}$ and a temperature of 35°C; thereby it can be seen that first IBs were monitored after NG= 0.1 indicating a rod shaped structure. Solely rod shaped IBs are present up until NG = 0.4, first potential sphere-like IBs might be observed at NG= 0.5 but still rod-shaped structures are present in dominant fraction for the ongoing induction time.

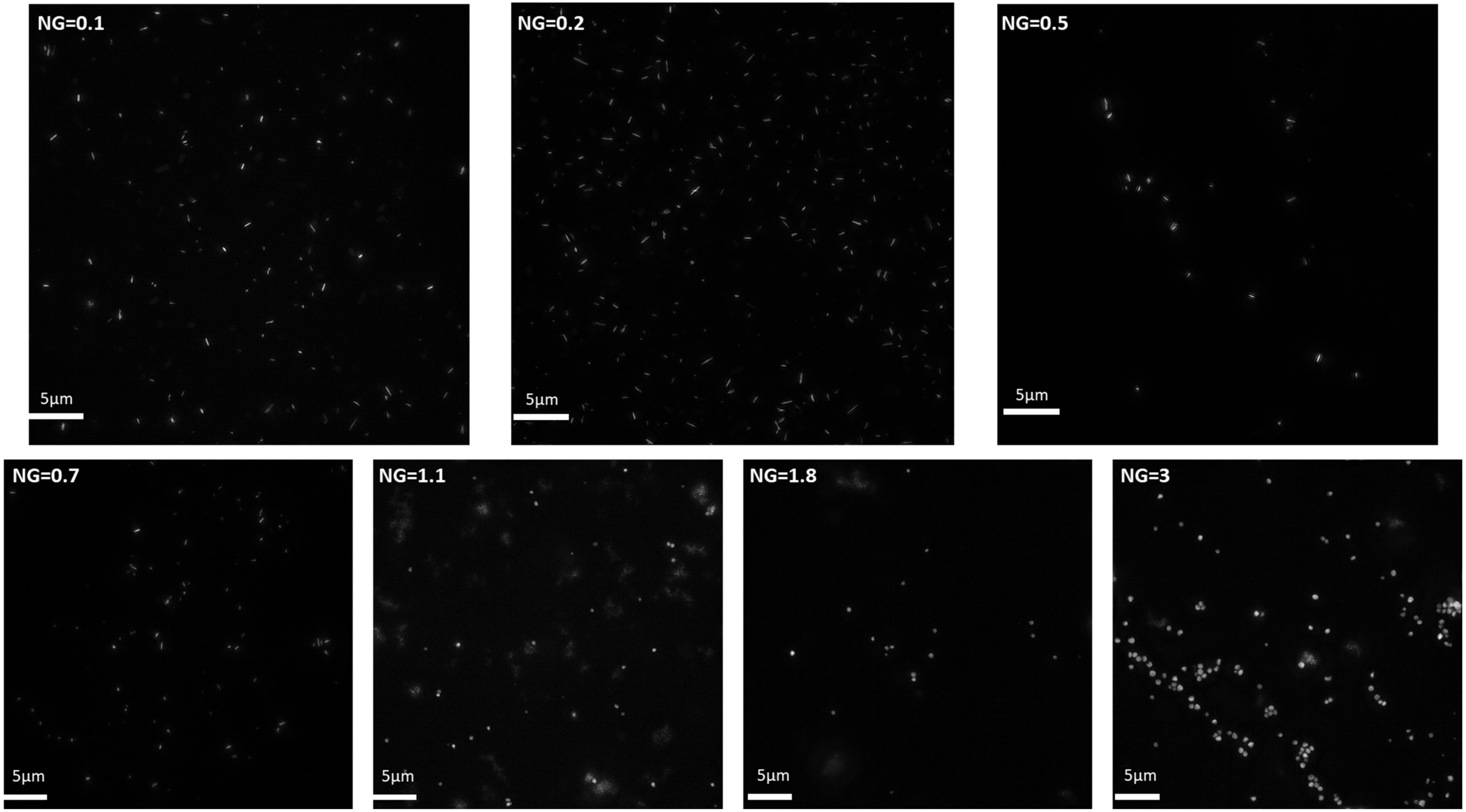

Supplementary Figure 4: Time-dependent OMX analysis of purified GFP IBs conducted for the cultivation operated at $\mu \sim 0.13\ h^{-1}$ and a temperature of 35°C; thereby it can be seen that rod shaped IBs are present until at least until NG= 0.7, however first sphere like structures can be also obtained at NG= 0.5. Solely spherical IBs are present from NG= 1.1 onwards and are present for the ongoing induction time, increasing in their size (Supplementary Figure 2).

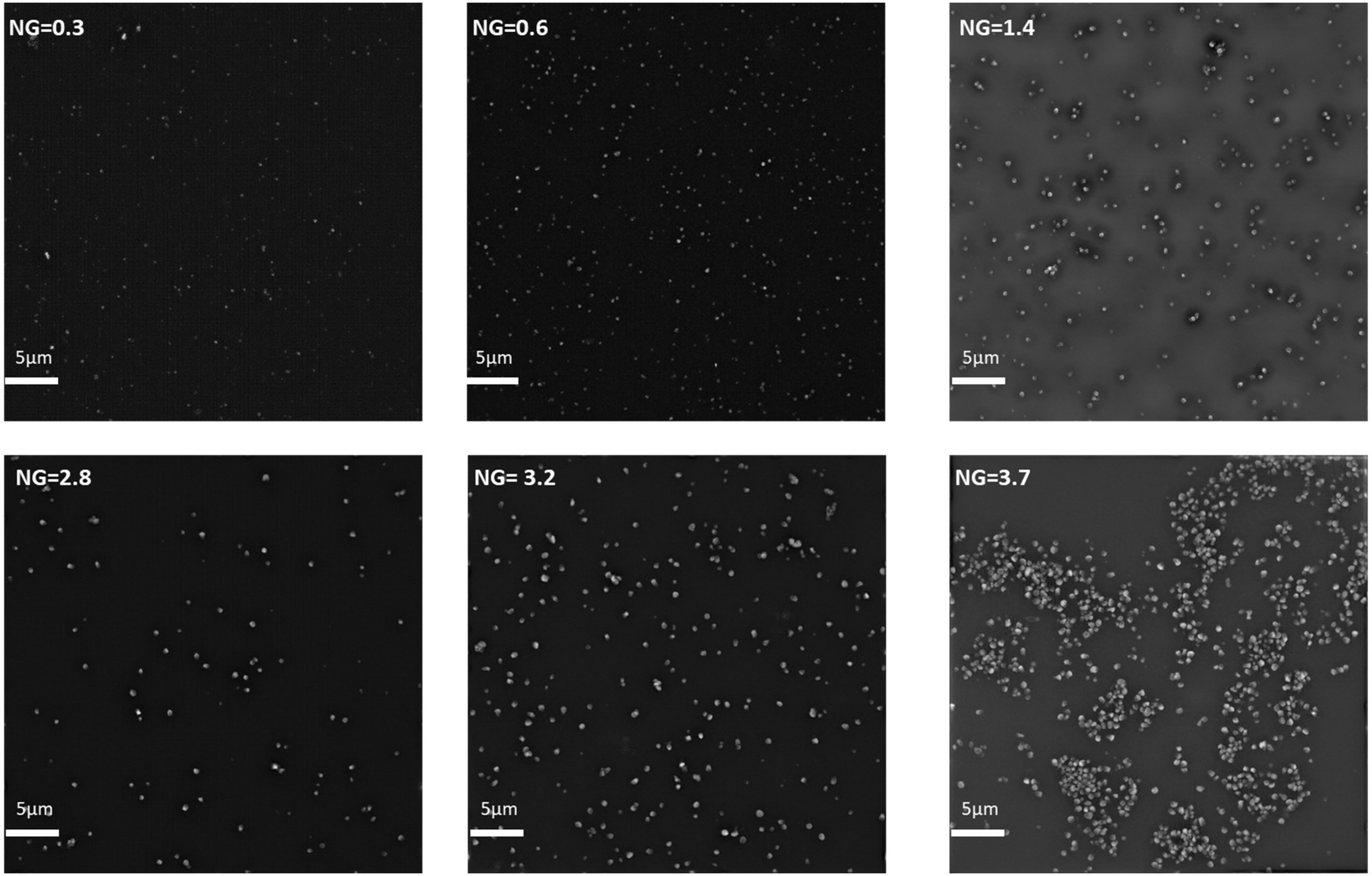

Supplementary Figure 5: Time-dependent OMX analysis of purified GFP IBs conducted for the cultivation operated at µ ~ 0.32 h-1 and a temperature of 35°C; thereby it can be seen that spherical IBs are present in dominant fraction already at NG=0.3, however also rod-shaped IBs can be seen. For NG beyond 0.6 solely spherical IBs can be seen, increasing in their size for the ongoing induction time. We attribute the faster sphere-like formation to a higher stress on the host cells due to the higher feeding rate applied, however effects of NG cab be still obtained.

Additional information on GFP+:

The gene was sequenced and showed 100% overlap with GFP[+] according to (Scholz, et al., 2000)

The amino acid sequence of this very protein is:

MSKGEELFTGVVPILVELDGDVNGHKFSVSGEGEGDATYGKLTLKFICTTGKLPVPW

PTLVTTLTYGVQCFSRYPDHMKRHDFFKSAMPEGYVQERTISFKDDGNYKTRAEVKF

EGDTLVNRIELKGIDFKEDGNILGHKLEYNYNSHNVYITADKQKNGIKANFKIRHNIE

DGSVQLADHYQQNTPIGDGPVLLPDNHYLSTQSALSKDPNEKRDHMVLLEFVTAAGI

THGMDELYKKXAAALEHHHHHH
